# TMPRSS2 as a Key Player in Viral Pathogenesis: Influenza and Coronaviruses

**DOI:** 10.3390/biom15010075

**Published:** 2025-01-07

**Authors:** Gilmara Barros de Lima, Everton Nencioni, Fábio Thimoteo, Camila Perea, Rafaela Fuzaro Alves Pinto, Sergio Daishi Sasaki

**Affiliations:** Graduate Program of Biosystems, Centro de Ciências Naturais e Humanas, Universidade Federal do ABC (UFABC), São Bernardo do Campo, São Paulo 09606-045, Brazil; lima.gilmara@ufabc.edu.br (G.B.d.L.); everton.nencioni@ufabc.edu.br (E.N.); fabio.mendonca@ufabc.edu.br (F.T.); camila.perea@ufabc.edu.br (C.P.); rafaela.fuzaro@aluno.ufabc.edu.br (R.F.A.P.)

**Keywords:** TMPRSS2, serine proteinase inhibitors, influenza, coronavirus

## Abstract

TMPRSS2, a human transmembrane protease enzyme, plays a crucial role in the spread of certain viruses, including influenza and coronaviruses. This enzyme promotes viral infection by cleaving viral glycoproteins, which helps viruses like SARS-CoV-2 and influenza A enter cells more effectively. Genetic differences in TMPRSS2 may affect people’s susceptibility to COVID-19, underscoring the need for studies that consider diverse populations. Beyond infectious diseases, TMPRSS2 has also been linked to some cancers, suggesting it could be a valuable target for drug development. This review provides a summary of TMPRSS2 inhibitors currently under study, with some already in clinical trials to test their effectiveness against viral infections. As we uncover more about TMPRSS2’s role in pathogenesis, it could open new doors for therapies to combat future outbreaks.

## 1. Introduction

The Human Transmembrane Protease Serine 2 (TMPRSS2) is a pivotal type II transmembrane protease, exhibiting widespread expression throughout the body. Initially described by Paoloni-Giacobino and colleagues in 1997, gene cloning efforts aimed at enriching the human transcriptome leveraged exons sourced from chromosome 21, meticulously charting pivotal genetic data underpinning this enzyme’s functionality [1]. TMPRSS2 is a member of the serine protease family, with genetic information located on chromosome 21 at position 21q22.3, comprising 14 exons and 13 introns [1,2]. Profiling its tissue expression pattern, TMPRSS2 is predominant in the heart, lungs, intestines, and prostate epithelium, and its transcription can be regulated by androgenic hormones [3,4,5,6].

**Figure 1:**
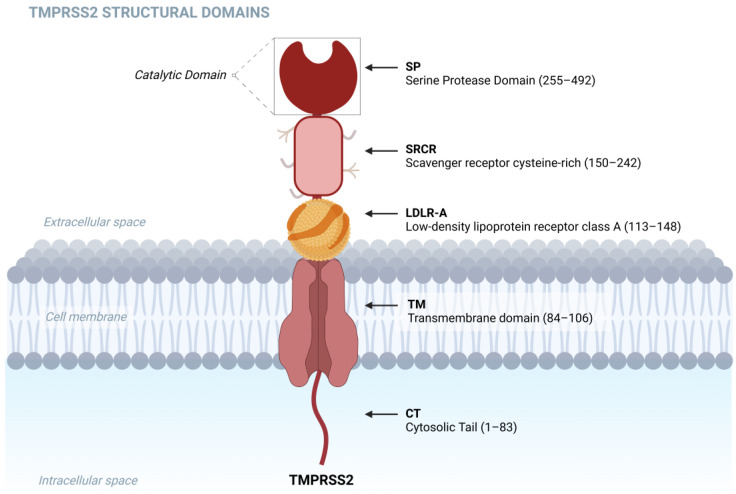
TMPRSS2 structural domains: cytosolic tail (CT, 1–83), and transmembrane domain (TM, 84–106). Low-density lipoprotein receptor class A (LDLR-A, 113–148), scavenger receptor cysteine-rich (SRCR, 150–242), and the serine protease domain (SP, 255–492). Created using BioRender. Barros de Lima, G. (2024) https://BioRender.com/x86m972, accessed on 15 November 2024.

The TMPRSS2 protein consists of 492 amino acids and is divided into five distinct domains. The first domain is the cytosolic tail (CT) intracellular domain (1–83), followed by the transmembrane domain (84–106). From amino acid 107 onwards, the protein adopts an extracellular conformation, with the presence of three additional domains: a short low-density lipoprotein receptor class A (LDLR-A, 113–148), a scavenger receptor cysteine-rich (SRCR, 150–242), and the serine protease domain (SP, 255–492) [1,7], which can be visualized in Figure 1.

The functions of each domain are as follows: the transmembrane domain provides stability to the phospholipid bilayer; the LDLR-A domain exhibits a structure similar to low-density cholesterol, forming binding bridges with lipidic and calcium sites; the SRCR domain plays a role in cell recognition, allowing interactions with specific proteins from other cell membranes; and, finally, the serine protease domain, an enzymatic region with trypsin-like proteolytic activity, is responsible for activating the SARS-CoV-2 spike (S) protein [8]. Although the biological functions of TMPRSS2 remain incompletely understood, it is well-established that this enzyme plays a vital role in activating the epithelial sodium channel (ENaC), which is essential for sodium transport in epithelial tissues, particularly in the airways [9]. There are also hypotheses suggesting that TMPRSS2 may be involved in protein digestion through cleavage when expressed in the intestine [10].

**Figure 2:**
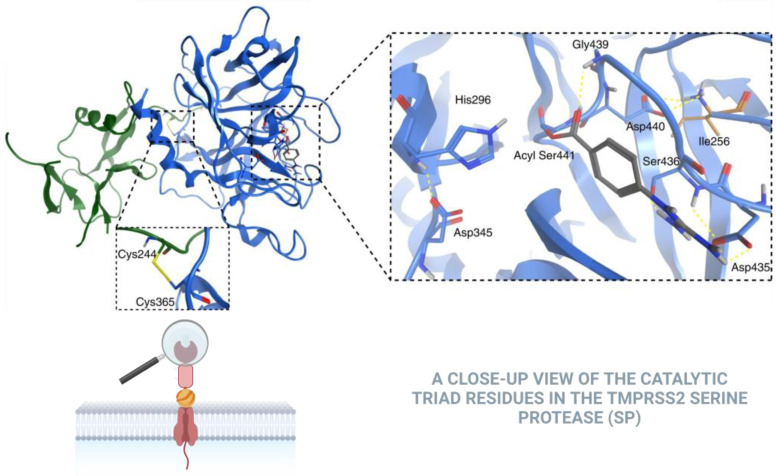
The X-ray crystal structure of TMPRSS2, treated with nafamostat, displays phenylguanidino acylation (illustrated by gray sticks), including His296, Asp345, and Ser441, and reveals the post-activation Asp440 salt bridge, indicating the complete maturation of the protease. The polar interactions are represented by yellow dashed lines. Additionally, the interdomain disulfide bond between Cys244 and Cys365 is crucial for maintaining the covalent linkage between the scavenger receptor cysteine-rich (SRCR) and serine protease domains. Adapted from Fraser et al., 2022 [8].

TMPRSS2’s structural and functional attributes further accentuate its significance. As shown in Figure 2 the TMPRSS2 SP domain is highly conserved within all type 2 transmembrane serine protease (TTSP) family members and conforms to the canonical chymotrypsin fold. The SP domain of TMPRSS2 uniquely possesses three disulfides and a single unpaired cysteine residue, Cys379, which is conserved in several species. TMPRSS2 recognizes and cleaves various protein substrates, particularly viral glycoproteins. The enzyme’s active site contains the S4-S3-S2-S1-S1′-S2′-S3′-S4′ subsites, which interact with substrate P4-P3-P2-P1↓P1′-P2′-P3′-P4′ amino acid positions spanning the scissile bond. The active site is characterized by a catalytic triad composed of three specific amino acids: histidine (296), aspartate (345), and serine (441). The S2 subsite includes a distinctive Lys342 residue, conferring a preference for small and/or electronegative P2 substrates [8]. Calcium binding is vital for regulating certain members of the TMPRSS family. However, human TMPRSS2 lacks the crucial Asp residue necessary for calcium chelation, indicating its inability to bind calcium at this site [6].

TMPRSS2 may also influence sodium control in cells by cleaving sodium channels in the plasma membrane, thereby facilitating ion passage. This occurs through the proteolytic cleavage of the γ subunit of ENaC, a critical step in enabling sodium ions to pass through epithelial cells. In human airway cells, the coexpression of TMPRSS2 with ENaC notably enhances ENaC-mediated currents by promoting channel opening. The catalytic activity of TMPRSS2 is essential, as inactive TMPRSS2 cannot activate ENaC. Fully cleaved ENaC is observed both on the cell surface and intracellularly, suggesting that TMPRSS2 is involved in ENaC activation before surface localization. Blocking a release mechanism in ENaC abolishes TMPRSS2’s effect, which is reinstated upon bond reduction. TMPRSS2 cleaves ENaC at multiple sites, and reducing TMPRSS2 expression results in incomplete ENaC activation. These findings not only clarify TMPRSS2’s role in regulating sodium transport but also highlight its potential relevance for respiratory diseases. For instance, in acute respiratory distress syndrome (ARDS), dysfunction in ENaC activation can impair sodium absorption and disrupt pulmonary fluid homeostasis. Therefore, the cleavage of ENaC by TMPRSS2 is crucial not only for normal physiological processes but also for understanding pathological conditions, particularly those impacting respiratory function [11].

Moreover, the alternative splicing of TMPRSS2 mRNA may produce a second isoform, suggesting functional diversity. Notably, the microRNA miR-98-5p has emerged as a significant regulator of TMPRSS2 gene transcription in specific cellular contexts, acting as a suppressor of TMPRSS2 transcription in human lung and umbilical vein endothelial cells [12].

The TMPRSS2 protease has garnered significant attention in scientific literature due to its pivotal role as a host cell factor facilitating the viral entry and pathogenesis of SARS-CoV-2, the causative agent of COVID-19 [13]. However, previous research has also elucidated TMPRSS2’s significance as an essential enzyme involved in cleaving hemagglutinin of various influenza virus subtypes, with its deficiency offering protection against specific influenza A virus (IAV) infections [14]. As investigations into the intricate interplay between TMPRSS2 and viral infections continue, a comprehensive understanding of its multifaceted functions holds promise for the development of innovative therapeutic strategies and a more nuanced approach to managing viral respiratory diseases.

In addition to its well-established role in respiratory viral infections, TMPRSS2 has been implicated in other infections, such as hepatitis C virus (HCV). Studies have demonstrated TMPRSS2’s activation of HCV infection, suggesting its significance in viral pathogenesis [15]. Furthermore, investigations into the TMPRSS2 expression in hepatocytes from chronic hepatitis B (CHB) and hepatocellular carcinoma (HCC) patients revealed markedly elevated levels in HCC tissues compared to para-carcinoma tissues. This finding underscores TMPRSS2’s involvement in hepatitis C and its potential as a therapeutic target for antiviral development [16]. Additionally, emerging research has linked TMPRSS2 to the pathogenesis of ulcerative colitis, expanding its relevance beyond viral infections [17]. Furthermore, TMPRSS2’s association with oncological diseases, including colorectal, gastric, prostatic, pulmonary, and hepatic cancers, has been extensively documented. The heightened gene expression of TMPRSS2 in these cancers suggests its potential as a biomarker and therapeutic target. Notably, patients with these malignancies are identified as a significant risk group due to their heightened susceptibility to viral contagion, underscoring the complex interplay between TMPRSS2, viral infections, and cancer [18].

## 2. Role of TMPRSS2 in Influenza Infections

Influenza represents an infectious disease induced by influenza viruses. These viruses are classified into four categories: A (genus *influenzavirus* A), B (genus *influenzavirus* B), C (genus *influenzavirus* C), and D (genus *influenzavirus* D). In the context of human seasonal epidemics, IAV and IBV emerge as the predominant types. In particular, IAVs are stratified into subtypes, distinguished by specific configurations of their surface hemagglutinin (HA) and neuraminidase (NA) proteins [19,20,21]. This categorization identifies 18 distinct HA subtypes and 11 diverse NA subtypes capable of rearranging freely, with many of the 144 potential variants observed in natural reservoirs. The interaction between HA and NA is essential for viral infectivity, which likely explains why certain combinations of HA and NA appear more frequently than others [22]. Currently, prevalent influenza A subtypes in human circulation include H1N1 and H3N2 [23]. Some influenza A viruses originating from animals have the ability to infect humans, leading to severe illnesses. Viruses such as H5N1 and H5N2 have recently been confirmed to infect humans, raising significant public health concerns [24,25]. In contrast, IBVs are not classified into subtypes but are instead categorized into distinct lineages: B/Yamagata and B/Victoria [26]. It is noteworthy that influenza C virus is less commonly detected and typically results in mild infections, thereby lacking significant public health implications [27].

**Figure 3:**
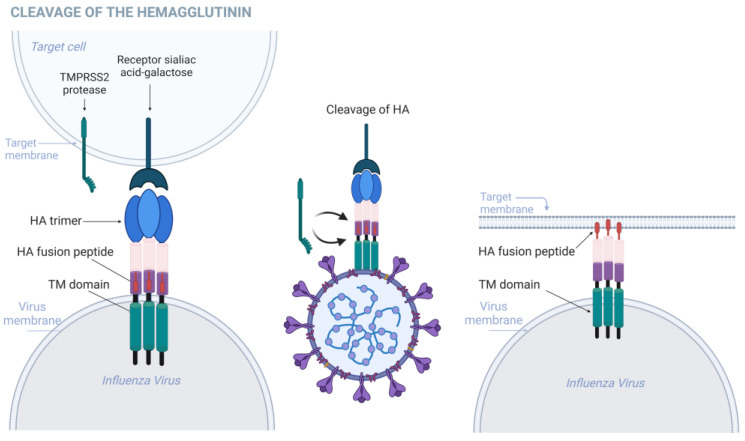
Cleavage of the hemagglutinin (HA) glycoprotein promotes virion attachment to the host cell. Created using BioRender. Lima, G. (2024) https://BioRender.com/d82o124, accessed on 15 November 2024.

Enveloped viruses gain entry into host cells through a process characterized by the binding of virions to cell surface receptors, followed by fusion with the host cell membrane. Viral envelope glycoproteins play a crucial role in regulating these processes. Specifically, in the context of influenza infections, HA is an antigenic glycoprotein present in various subtypes of IAVs. HA is responsible for both attaching the virus to cell surface receptors and facilitating the release of the viral genome into the cytoplasm through membrane fusion, as illustrated in Figure 3. HA is initially synthesized as an inactive precursor known as HA0 [28,29]. TMPRSS2 catalyzes the cleavage of HA0, resulting in two functional subunits: HA1 and HA2; this cleavage of HA0 resulting in disulfide-bound HA1 and HA2 subunits is a crucial step for virus infectivity, as it endows HA with fusion competence into HA1 and HA2 [14,30].

HA1 contains the receptor-binding domain responsible for attaching the virus to sialic receptors on host cell surfaces, while HA2 mediates membrane fusion [31]. Following proteolytic cleavage, HA1 and HA2 assemble into a homotrimeric structure. The HA1 subunit forms a globular “head” domain positioned atop the stem, while the three HA2 subunits collectively form the stem, enveloped by the N- and C-terminal segments of HA1. The liberation of the fusion peptide from the hydrophobic pocket of HA2 allows it to be inserted into target membranes during fusion. Additionally, the cleavage of precursor HA enables HA to adopt a metastable conformation that can be triggered by the acidic pH of endosomes. In response to acidic pH, HA undergoes structural rearrangements necessary for membrane fusion. The receptor-binding subdomain of HA1 attaches to sialic receptors on the target membrane surface, triggering virion internalization via the endocytic pathway. During endosome maturation, the acidic environment triggers further rearrangements in HA, leading to the full exposure and release of the fusion peptide from the inner pocket. Low pH-triggered conformational changes in HA2 cause fusion peptide insertion into the target endosomal membrane, forming an intermediate structure known as the pre hairpin. Several pre hairpins undergo further structural rearrangements, inducing hemifusion, and complete fusion, and ultimately generating a fusion pore, allowing viral genetic material to escape into the cytosol [32].

HA is composed of various subtypes and isoforms, each with unique traits influencing host specificity, virulence, and transmissibility [33]. Each subtype has a specific distribution across animal species and can adapt to different hosts. For instance, H1 is predominant in human and swine influenza, while H5 is often linked to avian influenza outbreaks and can, in some cases, infect humans [34]. The diversity of HA subtypes arises from genetic mutations and rearrangements, affecting HA’s receptor affinity, host range, and infectivity across species. For example, structural studies reveal that H5 has a higher affinity for α2,3-galactose sialic acid receptors, mostly in birds’ upper respiratory tracts, while H1 prefers α2,6-galactose sialic acid receptors, commonly found in human epithelial cells. HA isoforms also contribute significantly to influenza virus biology. Through alternative splicing and post-translational modifications, a single HA molecule can produce multiple isoforms with varying receptor affinities, stability, and immune evasion capabilities. Understanding HA’s structural and functional characteristics is essential for developing effective influenza control strategies, such as vaccines targeting prevalent subtypes. The continuous monitoring of the HA subtype and isoform evolution in animal and human populations is critical for detecting and quickly responding to new influenza outbreaks and pandemics [25,33,34].

It has been observed that TMPRSS2 activates the HA in IAV subtypes H1, H2, H3, H5, H6, H8, H10, H11, H14, and H15 [29]. In animal models, the deletion of the TMPRSS2 gene in mice has been shown to protect the host against viral spread in infected lungs, indicating the significance of TMPRSS2 in the pathogenesis of H1N1, H3N2, and H7N9 influenzas [35,36,37]. Wild-type mice exposed to H1N1 influenza virus exhibited a mortality rate of 20%, while those infected with H7N9 influenza virus experienced a strikingly higher mortality rate of 100%. Notably, in contrast to these outcomes, TMPRSS2-deficient mice infected with either H1N1 or H7N9 influenza viruses demonstrated apathogenic responses, suggesting a crucial role of TMPRSS2 in the virulence and pathogenicity of these viral strains [37]. In TMPRSS2 knock-out (KO) mice infected with low-pathogenicity influenza A viruses (LP IAVs), the cleavage of HA was significantly compromised, leading to a substantial reduction in infectivity for the majority of LP IAV progeny particles [38]. Experimental investigations involving TMPRSS2−/−-deficient mice and cell cultures have revealed the pivotal role of this host protease in facilitating the effective infection and dissemination of the IAV subtype H10 and H2 [38,39]. This emphasizes the critical function of TMPRSS2 in facilitating the cleavage of HA in vivo, underscoring the vital significance of TMPRSS2 expression for the proficient replication of IAV within an in vivo setting.

In vitro experiments demonstrated that TMPRSS2 is involved in the activation of IAV and IBV viruses. In a study, TMPRSS2 was identified as the major HA-activating protease of IAV in human airway cells and IBV in type II pneumocytes. The research explores the role of TMPRSS2 in the proteolytic activation of IAV and IBV, employing three distinct human airway cell types: primary human bronchial epithelial cells (HBECs), primary type II alveolar epithelial cells (AECIIs), and Calu-3 cells. To elucidate the impact of TMPRSS2, the study employed a targeted knockdown approach utilizing an antisense peptide-T-ex5. This peptide interferes with the splicing of TMPRSS2 pre-mRNA, leading to the expression of enzymatically inactive TMPRSS2. The efficiency of TMPRSS2 knockdown was evaluated across the three airway cell cultures. Notably, TMPRSS2 knockdown using T-ex5 resulted in a significant reduction of active TMPRSS2 in all tested cell types. Furthermore, the study investigated the consequences of TMPRSS2 knockdown on influenza virus activation. In HBEC and AECII, TMPRSS2 knockdown efficiently inhibited the proteolytic activation of both H7N9 and H3N2 IAV strains. Additionally, in AECII, the knockdown of active TMPRSS2 by T-ex5 hindered the proteolytic activation of IBV, leading to the expression of enzymatically inactive TMPRSS2 [40].

The influence of TMPRSS2 on the replication of pandemic H1N1 and H3N2 subtype IAVs was also investigated in the pig, a natural host for these viruses. Findings from in vitro studies showcased a delay in virus replication kinetics within primary bronchial epithelial cells sourced from TMPRSS2 KO pigs in comparison to their wild-type (WT) counterparts, albeit ultimately reaching comparable peak virus titers. Remarkably, infected KO pigs demonstrated minimal virus shedding from the nasal cavity, contrasting with the continuous shedding observed in WT pigs. Furthermore, IAV infection in KO pigs resulted in a decreased viral burden and less severe pathology in bronchoalveolar lavage fluid (BALF) compared to WT pigs. Unlike mice, which often fail to shed virus, TMPRSS2 KO pigs, as natural hosts for IAVs, exhibited impaired replication and a reduced pathology in both upper and lower respiratory tracts, underscoring the pivotal role of TMPRSS2 in virus replication and transmission. The study posits potential commercial applications of TMPRSS2 KO pigs in alleviating economic losses and mitigating the risk of emerging novel influenza viruses [41].

In the context of influenza C (ICV) and D (IDV) virus, the glycoprotein governing attachment and fusion with the host cell membrane is the hemagglutinin-esterase (HE), melding the functionalities of the HA and NA proteins present in IAV and IBV strains. This amalgamation underscores the complexity within viral taxonomy, delineating six distinct lineages for influenza C (C/Taylor, C/Yamagata, C/São Paulo, C/Aichi, C/Kanagawa, and C/Mississippi) [42], and five for influenza D (D/OK, D/660, D/Yama2016, D/Yama2019, and D/CA2019) [43], as deciphered from the HE gene sequences. Similar to HA, the proteolytic cleavage of HE involves TMPRSS2 [44,45].

In addition to its role in influenza, TMPRSS2 extends its influence to the activation of other respiratory viruses. Parainfluenza viruses [46] and human metapneumovirus (HMPV) effectively utilizes TMPRSS2 within the human lung epithelium to cleave its F protein, thus facilitating viral replication [47,48]. However, undoubtedly the most extensively researched role of TMPRSS2 lies in its involvement with coronaviruses.

## 3. Role of TMPRSS2 in Coronavirus Infections

The TMPRSS2 enzyme was first identified as a host cell factor essential for viral spread and pathogenesis during the 2002–2003 outbreak of Severe Acute Respiratory Syndrome Coronavirus (SARS-CoV). It is involved in cleaving the viral spike (S) protein, which is essential for viral entry into host cells [49,50,51]. Apart from its involvement in SARS-CoV, TMPRSS2 is also significant in the pathogenesis of the Middle East Respiratory Syndrome Coronavirus (MERS-CoV), which emerged in Saudi Arabia in 2012 [52,53]. Studies using animal models infected with MERS-COV found that TMPRSS2 deficiency changes viral kinetics and reduces immunopathological responses. The absence of the TMPRSS2 protease in murine models resulted in a significant reduction in the severity of lung pathology following SARS-CoV and MERS-CoV infection. Specifically, C57BL/6 mice, prone to a Th1-type immune response, and TMPRSS2 KO mice were used for SARS-CoV infection, while transgenic mice expressing the human DPP4 receptor for MERS-CoV (hDPP4-Tg) and hDPP4-Tg KO mice for TMPRSS2 were employed for MERS-CoV infection. After experimental infection, TMPRSS2-deficient strains exhibited less body weight loss and reduced viral kinetics in the lungs. The absence of TMPRSS2 affected key sites of viral infection and dissemination in the airways, accompanied by less severe immunopathology. However, TMPRSS2-KO mice displayed attenuated inflammatory chemokine and cytokine responses upon intranasal stimulation with poly (I·C), a Toll-like receptor 3 agonist. This underscores TMPRSS2’s pivotal involvement in viral dissemination and pathogenesis [54].

Moreover, TMPRSS2’s influence extends to HCoV-229E, a human coronavirus linked to common cold and pneumonia, particularly in immunocompromised individuals, as well as in human coronavirus HKU1, a β-coronavirus known to circulate in humans seasonally [50,55]. In HKU1, TMPRSS2 acts as a functional receptor alongside sialoglycans, priming the viral spike protein for membrane fusion. The serine peptidase domain of TMPRSS2 is essential for HKU1 entry [56], and its autocleavage enhances the binding affinity to the spike protein, facilitating effective membrane fusion [57,58]. While TMPRSS2’s involvement in various coronaviruses has been explored, its most extensively researched role undoubtedly lies in its connection with the global health crisis caused by Severe Acute Respiratory Syndrome Coronavirus 2 (SARS-CoV-2).

As soon as COVID-19 emerged on the global stage and became a pandemic, resulting in significant loss of life, scientific communities worldwide swiftly directed their focus towards elucidating the intricate infection mechanisms inherent to the SARS-CoV-2 virus in hopes of finding solutions to stop the spread of the pandemic. It was previously established that SARS-CoV viruses utilize two distinct pathways for cellular entry: one mediated by TMPRSS2 on the cell surface and the other facilitated by cathepsin L/B within the endosome [59,60]. Thus, existing knowledge underscores the pivotal role of TMPRSS2 in activating this viral group through proteolysis, highlighting its significance as a focal point for COVID-19-related investigation [61,62,63,64].

The SARS-CoV-2 virus expresses a spike (S) protein, a class I fusion protein that assembles into a trimer and consists of two key functional subunits: the S1 subunit, which facilitates receptor binding, and the S2 subunit, responsible for membrane fusion [61,65]. This spike protein is heavily glycosylated and these glycans play a crucial role in the overall structure and stability of the spike protein and may also influence immune recognition and viral infectivity [66]. The trimeric arrangement of the spike protein facilitates efficient receptor binding and subsequent membrane fusion, enabling viral entry into host cells [67,68]. Moreover, the spike protein belongs to the class I viral fusion proteins, sharing structural similarities with influenza HA. Consequently, both coronavirus S proteins and influenza HA undergo conformational transitions that promote membrane fusion [69].

**Figure 4:**
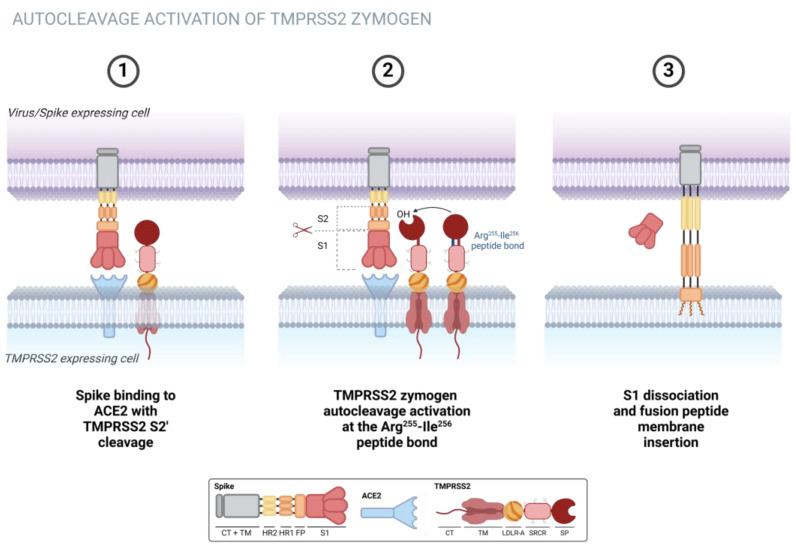
The membrane-bound TMPRSS2 zymogen experiences autocleavage activation at the Arg255-Ile256 peptide bond, leading to the formation of the mature enzyme. Subsequently, the matured enzyme proteolytically cleaves the SARS-CoV-2 spike protein (shown in magnification) attached to the ACE2 receptor (depicted in yellow), facilitating membrane fusion. Created using BioRender. Lima, G. (2024) https://BioRender.com/u69o481, accessed on 15 November 2024.

In the context of COVID-19 infections, SARS-CoV-2 initially attaches to host cells via S protein binding to angiotensin-converting enzyme 2 (ACE2) receptors on the cell surface. However, for entry into host cells, the virus relies on the cleavage of the spike protein by the host cell protease TMPRSS2. This cleavage event promotes the activation of the S protein, enabling membrane fusion and the subsequent infection of the host cell [3,61,70], as shown in Figure 4.

Experimental investigations have also elucidated TMPRSS2’s regulatory mechanisms, including its autoactivation via intracellular autocatalysis and the importance of N-glycosylation for proper protein folding and trafficking. In experiments conducted with human embryonic kidney 293 (HEK293), bronchial epithelial 16HBE, and lung alveolar epithelial A549 cells, it was discovered that TMPRSS2 undergoes autoactivation through intracellular autocatalysis in the Golgi apparatus after leaving the endoplasmic reticulum (ER) [64]. This process is effectively inhibited by a natural inhibitor known as HAI-2 [71]. Furthermore, researchers identified N-glycosylation at the sites N250 and N286. The N-glycan at N250 in the SRCR domain aids in calnexin-mediated folding and ER exiting, impacting intracellular trafficking, zymogen activation, and cell surface expression. ER retention of the N250Q mutant suggests impaired zymogen activation. Treatment with deoxynojirimycin (DNJ), inhibiting calnexin-N-glycan interaction, causing the blocked activation of the TMPRSS2 WT and N286Q mutant, supporting the role of N-glycosylation in TMPRSS2 activation. Remarkably, TMPRSS2 was observed to cleave SARS-CoV-2 S protein intracellularly within HEK293 cells, indicating its potential involvement in viral entry beyond just the cell surface [64]. These findings significantly improve our understanding of TMPRSS2’s regulatory mechanisms and underscore its importance in respiratory viral infections.

SARS-CoV-2 primarily relies on TMPRSS2 rather than cathepsins for cell entry [60,61,72]. Research on interferon-induced transmembrane proteins (IFITM), which are endosomal virus restriction factors induced by type I interferon, provides further insights. SARS-CoV-2 lacking the polybasic site is more susceptible to IFITM-mediated restriction compared to the wild-type virus, suggesting that the virus utilizes TMPRSS2 to evade the IFITM-mediated restriction in the endosome. Alternatively, the preference of SARS-CoV-2 for TMPRSS2 may be attributed to differences in S protein folding. The tight folding of the SARS-CoV S protein ensures the precise exposure of the S2′ site to cathepsin L. In contrast, variations in SARS-CoV-2 S protein folding may result in the excessive digestion of neighboring sequences, rendering the fusion peptide non-functional and indicating the preference for TMPRSS2 over cathepsins [73,74,75].

Furthermore, a study investigates the spatial distribution of ACE2 and TMPRSS2 expression in the lung lobes of Syrian hamsters and its influence on the infection patterns of SARS-CoV-2. The research reveals distinct arrangements of ACE2 and TMPRSS2 expression across the lung lobes, which contribute to variations in the susceptibility and severity of SARS-CoV-2 infection. The findings indicate that infection occurs primarily in areas where both ACE2 and TMPRSS2 are present. TMPRSS2 expression is notable in the primary and secondary bronchi, while ACE2 is predominantly observed in the bronchioles and alveoli. However, infection fully overlaps in regions where ACE2 and TMPRSS2 co-localize, specifically in the tertiary bronchi, bronchioles, and alveoli. This spatial heterogeneity suggests that specific lung regions may serve as preferential sites for viral entry and replication, influencing the pathogenesis and spread of the virus within the respiratory system [76].

While the catalytic domain of TMPRSS2 is well-documented for its role in mediating viral entry, other regions of TMPRSS2 have received less attention. Through phosphomimetic mutation experiments, the TMPRSS2 cytosolic tail is identified as a potential substrate for phosphorylation by tyrosine kinases, demonstrating that phosphorylation can downregulate TMPRSS2 enzymatic activity. This suggests that targeting TMPRSS2 phosphorylation could offer a virus-specific approach for antiviral strategies. There is a potential association between a TMPRSS2 gene single-nucleotide polymorphism (SNP) causing a V160M mutation and severe COVID-19 disease progression. A spike-binding region within the TMPRSS2-SRCR domain suggests the existence of a second binding region. Furthermore, the role of the H655 residue in mediating the TMPRSS2 interaction with the SARS-CoV-2 spike protein and the affinity between the TMPRSS2 and spike affect the infection pathway via the cell membrane, potentially reducing the utilization of the endosomal route for viral entry [77].

A topic of debate has been the dependence of the Omicron variant on TMPRSS2 for host cell entry. While some studies suggest that Omicron relies less on TMPRSS2 compared to earlier variants like Delta, others highlight its continued role in certain contexts. Research shows that Omicron exhibits a lower dependency on TMPRSS2, particularly in pediatric populations where the TMPRSS2 expression is reduced [78], which may partly explain its rapid spread among children. Despite these findings, TMPRSS2 still appears to facilitate aspects of Omicron infectivity in tissues with a high TMPRSS2 expression [79]. These contrasting observations underscore the complexity of Omicron’s entry mechanisms and the need for further investigation.

Recently, evidence has emerged indicating that BANAL-CoVs (bat coronaviruses), considered the closest known relatives of SARS-CoV-2, also utilize TMPRSS2 for host cell entry [80]. This finding emphasizes TMPRSS2’s pivotal role in enabling coronavirus infections and its potential involvement in zoonotic transmission.

It is noteworthy that TMPRSS2 is an androgen-responsive serine protease, with its expression extending beyond the confines of lung tissue. Its predominant presence in prostate epithelium underscores its pivotal role in prostate carcinogenesis [81]. But, beyond the prostate, TMPRSS2 finds expression in varied tissues such as the cardiac endothelium, kidneys, and digestive tract, suggesting the potential susceptibility to SARS-CoV-2 infection in these organs [82]. Indeed, the clinical spectrum of COVID-19 encompasses not only respiratory complications but also extrapulmonary manifestations, including myocardial injury, liver enzyme elevation, acute kidney injury, and gastrointestinal symptoms. TMPRSS2’s presence in microvascular endothelial cells adds another layer of complexity, as SARS-CoV-2 infection could instigate endothelial dysfunction, predisposing individuals to thrombotic events and associated complications [83]. Examining autopsy tissues of COVID-19 patients, it becomes evident that there is a notable upregulation of ACE2 expression, along with the presence of TMPRSS2 and the consequential endotheliitis in capillaries [84,85,86,87]. These observations shed light on TMPRSS2’s role in the pathophysiology of COVID-19, offering insights into the diverse clinical manifestations observed in patients with severe disease. Understanding the intricate interplay between TMPRSS2 expression and SARS-CoV-2 infection holds promise for elucidating disease mechanisms and developing targeted therapeutic interventions.

The multifaceted role of TMPRSS2 in coronavirus infections, particularly its involvement in viral entry, the regulation of infection pathways, and its interplay with host genetic factors, underscores its significance as a potential therapeutic target and highlights the need to better understand its implications in disease pathogenesis.

## 4. TMPRSS2 Polymorphisms and COVID-19

Research into COVID-19 severity has revealed a significant correlation with genetic factors. A comprehensive study identified 551 single-nucleotide variations (SNVs) within the TMPRSS2 gene by analyzing a large cohort of 156,513 individuals. These SNVs are distributed across the gene’s coding regions, with the majority being rare (allele frequency <0.1%) and population-specific. Notably, certain variants are computationally predicted to be more likely deleterious than those with more common allele frequencies, suggesting a potential impact on the susceptibility to diseases, including COVID-19 [88]. This complex genetic landscape helps explain the observed differences in TMPRSS2 genotype distributions between patients with mild and severe COVID-19.

TMPRSS2 polymorphisms, particularly the rs12329760 variant (p.Val197Met), have drawn attention because their role in various health conditions, including the susceptibility to SARS-CoV-2 and prostate cancer. A study found the rs12329760 polymorphism to be closely linked to disease severity, with the T allele identified as a significant risk marker. The rs17854725 A > G polymorphism was also associated with a more aggressive disease presentation [89].

Another study involving 609 COVID-19 patients confirmed by RT-PCR and 291 SARS-CoV-2-negative individuals, identified four TMPRSS2 gene polymorphisms (rs12329760, rs2298659, rs456298, and rs462574) as contributing to disease severity and mortality. Using 5′ exonuclease TaqMan assays, the study found the rs12329760 T allele to be linked to more severe COVID-19 outcomes. These findings underscore the genetic contribution of TMPRSS2 to COVID-19 severity and susceptibility across different populations [90].

Further research identified the rs2070788 GG genetic mutation, which leads to a higher TMPRSS2 protein expression in lung tissue, as correlated with a significant increase in mortality risk among elderly COVID-19 patients [91].

Population differences also reveal intriguing insights. The TT genotype of TMPRSS2 was significantly more frequent in severe COVID-19 cases, highlighting a strong association with an increased susceptibility to severe disease [92,93]

Curiously, the healthcare experience among Indigenous communities in the Brazilian Amazon region, particularly in the state of Pará, revealed a different result. Despite the rapid spread of SARS-CoV-2, Indigenous peoples in Brazil largely experienced asymptomatic or mild COVID-19 cases, with mortality rates significantly lower compared to the broader Brazilian population. In 2020, prior to vaccination efforts, a notable prevalence of IgG anti-SARS-CoV-2 antibodies was detected among these individuals, suggesting that they reached collective immunity levels within a few months of the infection’s onset. Although the study examined genetic factors, including genotyping the TMPRSS2 rs123297605 C/T variant, no direct association was found between this variant and the presence of IgG antibodies, which indicates an immune response to the virus. The study did not observe a strong correlation between genetic variations and disease severity, but it is possible that Indigenous populations possess protective genetic variants that reduce their susceptibility to severe COVID-19 outcomes. Furthermore, the research revealed that the frequency of risk alleles, such as the TMPRSS2 rs123297605 variant, was lower in Indigenous populations compared to continental and other Brazilian populations. These findings underscore the importance of considering ethnic-specific genetic factors in understanding disease susceptibility and severity, offering valuable insights into the disparities observed in COVID-19 outcomes among different populations [94].

The T allele of TMPRSS2 rs12329760 has been associated with lower-disease-severity patient categories, indicating a protective effect against severe disease [95]. A substantial difference in the distribution of TMPRSS2 genotypes was observed between mild and severe COVID-19 cases, with the C allele being more prevalent in severe cases (91.7%) compared to mild cases (22.2%) [96]. Table 1 presents a summary of the TMPRSS2 gene variants studied in association with COVID-19 severity.

The association between TMPRSS2 alleles and COVID-19 severity is indeed a topic of discussion in the scientific community. While many studies suggest that the minor T allele of the rs12329760 variant is protective against severe COVID-19, there are contrasting results from different regions like Iran and Egypt regarding the association of the T allele with severe COVID-19 cases [97]. Additionally, this variant is not linked to an increased risk of developing acute coronary syndrome in patients with COVID-19 [98].

These findings highlight the necessity of considering population-specific genetic factors in elucidating disease mechanisms and disparities in COVID-19 outcomes, enriching our understanding of the pathogenesis and potential therapeutic targets for combating the pandemic.

## 5. TMPRSS2 as a Potential Target for Treatment

TMPRSS2 has emerged as a key therapeutic target in the fight against respiratory viruses, particularly influenza and coronaviruses like SARS-CoV-2, garnering significant attention in recent research [99]. Consequently, TMPRSS2 inhibitors are seen as promising candidates for antiviral therapy and have been identified through both computational and experimental approaches. These inhibitors encompass a range of modalities, including small molecules, peptides, and proteins, and have shown promise in preventing viral entry for both influenza viruses and coronaviruses, as shown in Table 2. Several of these inhibitors are currently being evaluated in clinical trials as potential therapeutic agents.

Compounds such as camostat and nafamostat, which inhibit TMPRSS2 activity, have demonstrated potential in blocking viral entry mediated by the priming of the SARS-CoV-2 spike protein [115].

Camostat mesylate, an oral serine protease inhibitor targeting transmembrane protease serine 2 (TMPRSS2), has been approved for COVID-19 phase III trials. Originally approved in Japan in 1985 for treating pancreatitis due to its trypsin inhibitory activity, camostat gained attention in COVID-19 research for its potential to block TMPRSS2, as demonstrated in in vitro experiments [2]. Following these promising findings, Dr. Geoffrey Chupp, Director of the Yale Center for Asthma and Airways Disease, and Dr. Joseph Vinetz, Professor in the Section of Infectious Diseases, led a clinical trial to assess camostat’s efficacy in COVID-19 patients. This phase II randomized, double-blind, placebo-controlled trial included two groups—35 COVID-19 patients receiving 200 mg of camostat orally for seven days, and a placebo group. The results showed that the camostat group experienced a rapid resolution of COVID-19 symptoms, including an improvement in the loss of taste and smell. However, the RT-PCR testing of the nasopharyngeal SARS-CoV-2 viral load revealed no significant reduction in viral levels between the two groups. The study concluded in phase II in 2022 due to the inconclusive evidence of antiviral effects and the widespread impact of vaccine immunization [116]. Mechanistically, camostat inhibits TMPRSS2, blocking the proteolytic activation of the SARS-CoV-2 spike protein required for viral entry [104]. Despite this theoretical efficacy, a systematic review of randomized controlled trials found no significant clinical improvement or reduction in viral load among camostat-treated patients compared to placebo [117,118]. The evidence thus far remains inconclusive regarding its impact on mortality and recovery times. Ongoing research seeks to develop more potent TMPRSS2 inhibitors, as camostat’s limited plasma half-life may restrict its clinical effectiveness [103,119].

Nafamostat mesylate, originally approved in Japan in 1993 as an anticoagulant for surgical applications [120], was initially developed for treating pancreatitis and inhibiting TMPRSS2. Studies led by Gian Paolo Rossi at the University Hospital of Padova, structured as randomized, double-blind, placebo-controlled clinical trials, aim to evaluate nafamostat’s efficacy in COVID-19 patients [121]. Administered intravenously to hospitalized patients between the ages of 18 and 85 with oxygen saturation levels below 94%, the study assesses various clinical outcomes over a 28-day period, including the patient response rate, severity of illness, mortality, duration of hospitalization, cardiovascular events, and need for mechanical ventilation. Nafamostat has shown promise in preclinical models, demonstrating efficacy in reducing viral load and lung damage in SARS-CoV-2-infected animals [122]. Mechanistically, nafamostat effectively inhibits TMPRSS2, blocking the cleavage of the viral spike protein required for viral entry, and has demonstrated greater potency than camostat in human airway epithelial models [104]. Clinical trials, including the RACONA study, have shown a favorable safety profile, though results regarding mortality reduction in COVID-19 patients remain inconclusive [120]. Despite its potential, the clinical efficacy of nafamostat as a COVID-19 treatment remains uncertain, underscoring the need for further research.

Diminazene, a veterinary medicinal agent well-known as a furin inhibitor, also inhibited TMPRSS2 with an IC50 of 1.35 μM using novel cell-based assays. This makes diminazene a dual inhibitor of these two proteases [105].

Gabexate is a synthetic serine protease inhibitor primarily used to treat various forms of acute pancreatitis, including traumatic, edematous, and acute necrotizing pancreatitis. Known for its ability to inhibit TMPRSS2, gabexate has been investigated as a potential therapeutic agent for COVID-19 due to its antithrombin inhibitory properties. Its suppression of TMPRSS2 positions gabexate as a promising candidate for further research in antiviral treatments [123].

In one study, the trypsin-like serine protease inhibitors MI-432 and MI-1900, in combination with the furin inhibitor MI-1851, were shown to significantly reduce viral replication in vitro [34]. Furthermore, the TMPRSS2 inhibitor N-0385, a highly potent tetrapeptide compound featuring a ketobenzothiazole warhead, demonstrated efficacy against SARS-CoV-2 variants of concern—including Alpha, Beta, P1, and Delta—at nanomolar concentrations. Remarkably, N-0385 proved to be 10–20 times more potent than the TMPRSS2 inhibitor camostat against certain Omicron sub variants, highlighting its enhanced efficacy [110,124].

Omicsynin B4, a pseudo-tetrapeptide compound identified from *Streptomyces* sp. 1647, exhibits inhibitory activity against the host proteases cathepsin L and TMPRSS2. In a study, Omicsynin B4 demonstrated broad-spectrum anti-coronavirus activity against HCoV-229E, HCoV-OC43, and the SARS-CoV-2 prototype and its variants in multiple cell lines. Mechanistically, Omicsynin B4 blocked viral entry, which was supported by biochemical assays showing that Omicsynin B4 exhibited sub-nanomolar inhibition against cathepsin L and sub-micromolar inhibition against TMPRSS2. A molecular docking analysis confirmed that Omicsynin B4 binds effectively to the substrate binding sites of cathepsin L and TMPRSS2, forming covalent bonds. Therefore, Omicsynin B4 is a promising natural protease inhibitor that can block the entry of various coronaviruses [112].

DON and DRP-104, two additional serine protease inhibitors, exhibited potent inhibitory activity against TMPRSS2, comparable to or even exceeding that of clinically relevant inhibitors such as nafamostat. However, further in vitro and in vivo validation is still needed to fully assess their efficacy and safety as antiviral agents [125].

Additionally, natural compounds like ginsenosides have shown promise as TMPRSS2 inhibitors, with bioinformatics analyses revealing correlations between TMPRSS2 expression and immune cell recruitment in lung cancer patients, suggesting TMPRSS2 as a potential prognostic biomarker and immunomodulator target for combination therapies [126].

Targeting TMPRSS2 with inhibitors is advantageous as the TMPRSS2 cleavage site on the SARS-CoV-2 spike protein has remained highly conserved, suggesting these inhibitors may retain potency against future variants. Additionally, host-directed therapies like TMPRSS2 inhibition have a lower potential for resistance development compared to virus-directed therapies.

## 6. Clinical Trials

Clinical trials are pivotal in the evaluation of novel drugs, offering a structured and controlled setting to assess both the safety and efficacy of potential treatments. Volunteers, guided by a protocol outlining patient demographics, drug specifics, dosages, duration, and desired outcomes, partake in these trials. Through meticulous execution, researchers ascertain the safety and effectiveness of new drugs tailored to specific populations. Moreover, clinical trials facilitate the exploration of innovative methods to enhance the efficacy of standard treatments or mitigate side effects. Our search for TMPRSS2 inhibitors found 23 clinical trials registered in the ClinicalTrials.gov database. Of these, nine have an unknown status, eight are complete, and five are ongoing, as shown in Table 3.

The COMBO trial, conducted by the Sidney Kimmel Comprehensive Cancer Center at Johns Hopkins (ID NCT04652765), aims to assess the efficacy of combining camostat mesilate and bicalutamide in reducing the proportion of COVID-19 patients requiring hospitalization. This open-label study, initiated on 3 February 2021, enrolled six patients. In this Phase 1 interventional trial, patients diagnosed with symptomatic COVID-19 are randomized to receive either standard care alone or a combination of camostat mesilate and bicalutamide. A Phase 2 trial found that camostat mesilate helped patients recover from their symptoms faster and improved their sense of taste and smell, although it did not significantly reduce the viral load [116]. Additionally, the drug’s metabolite, GBPA, also exhibits antiviral properties, suggesting that camostat mesilate may have effects beyond its initial form [61,127]. In a Phase 3 study, patients treated with camostat mesilate did not experience a significant difference in viral clearance compared to those who received a placebo, with both groups taking an average of 11 days to test negative [128].

Meanwhile, the RACONA study (ID NCT04352400), sponsored by the University Hospital Padova, Italy, seeks to evaluate the efficacy of nafamostat mesylate in mitigating lung function deterioration and reducing the need for intensive care admission among COVID-19 patients. This double-blind, randomized placebo-controlled trial commenced on 4 June 2021, with an estimated completion by December 2024. With an enrollment target of 256 participants, this Phase 2/3 trial aims to elucidate the therapeutic potential of nafamostat, a potent TMPRSS2 inhibitor, in managing COVID-19-related lung complications, leveraging its anticoagulant and anti-pancreatitis properties observed in vitro and its approved use in cystic fibrosis treatment [111].

Together, the results of these trials could shape future treatment protocols, potentially expanding options for managing respiratory complications in COVID-19 and other viral infections.

## 7. Conclusions and Discussion

TMPRSS2 is a crucial enzyme for viral spread, especially through the proteolytic cleavage of viral glycoproteins. The genetic background of TMPRSS2, including its location on chromosome 21 and its role in the human transcriptome, is essential for understanding its influence on COVID-19 severity across different populations. Furthermore, the involvement of TMPRSS2 is not limited to viral infections but also extends to other diseases such as hepatitis C, ulcerative colitis, and several types of cancer, suggesting its potential as a therapeutic target.

Various inhibitors of TMPRSS2 have been researched, showing a high potency against certain viral subtypes. The development of therapies targeting this enzyme is particularly important in light of emerging variants resistant to current treatments, making it applicable to a wide range of viral infections beyond COVID-19.

This is especially because numerous investigations have documented instances of co-infection involving SARS-CoV-2 and IAV among patients diagnosed with COVID-19, with such co-infections frequently linked to severe respiratory complications and heightened mortality rates. For example, studies utilizing human-pluripotent-stem-cell-derived alveolar type II organoids (hiAT2) have shown that these cells are susceptible to infection by both SARS-CoV-2 and IAV. Interestingly, infection with one virus significantly increased the susceptibility to subsequent infection by the other virus. Specifically, SARS-CoV-2 Delta variants were found to upregulate α-2-3-linked sialic acid expression, while IAV infection led to the increased expression of angiotensin-converting enzyme 2 (ACE2) and TMPRSS2. Furthermore, co-infection with SARS-CoV-2 and IAV induced the hyperactivation of pro-inflammatory and immune-related signaling pathways, exacerbating cellular damage in hiAT2 organoids compared to infection with either virus alone [129]. This highlights the potential therapeutic significance of targeting TMPRSS2 to mitigate the impact of respiratory viral infections.

Investigating the TMPRSS2 enzymatic mechanism is important to designing and engineering new drugs that block and avoid virus initialization. More studies are necessary in order to understand deeply how the TMPRSS2 physiological process works and associate this with physiopathology, suggesting new clinical trials to discover new drug candidates.

## Figures and Tables

**Table 1:** Summary of TMPRSS2 gene variants and their association with COVID-19 severity.

Variant (SNP ID)	Genotype Allele	Associated Health Condition	Association with COVID-19 Severity	Ref.
rs12329760 (p.Val197Met)	T/C	COVID-19 Severity	T allele linked to increased severity	[89,90]
rs17854725	A/G	Aggressive COVID-19	G allele linked to severe COVID-19	[89]
rs2070788	G/G	Increased TMPRSS2 Expression in Lungs	GG genotype associated with higher mortality in elderly COVID-19 patients	[91]
rs462574	T/C	COVID-19 Severity	Significant association with severity	[90]
rs123297605	C/T	Asymptomatic or mild cases, collective immunity	Lower frequency of risk alleles, and lower mortality	[94]

**Table 2:** TMPRSS2 inhibitors.

Compound	Inhibition (Type)	Approval State	Ref.
Antithrombin	Irreversible	Phase II	[100]
Aprotinin	Competitive	Phase III clinical trial	[101]
Argatroban	Competitive	Phase IV	[102]
BC-11	Irreversible	Preclinical Phase	[103]
Camostat Mesylate	Irreversible	Phase III COVID-19, 2021	[104]
Diminazene	Competitive	In vitro assay	[105]
Famotidine	Competitive	Phase IV	[102]
Gabexate	Competitive	Preclinical Phase	[62,106]
Guanadrel	Competitive	In silico simulation	[102]
Guanethidine	Competitive	In silico simulation	[102]
MI-485, MI-472, MI-1900, MI-1903, and MI-1904	Competitive	In vitro assay	[107,108]
MI-463	Not determined	In vitro assay	[108]
Narigin	Competitive	In silico simulation	[109]
N-0385	Irreversible	Animal model	[110]
Nafamostat	Irreversible	Phase III COVID-19, 2020	[111]
Neohesperidin	Competitive	In silico simulation	[109]
Omicsynin B4	Irreversible	In vitro assay	[112]
Otamixaban	Competitive	Preclinical Phase	[113]
Rhoifolin	Competitive	In silico simulation	[109]
Vicenin-2	Competitive	In silico simulation	[109]
α1-antitrypsin	Irreversible	In vitro assay	[114]

Inhibitors listed have shown potential for blocking TMPRSS2 activity in various preclinical studies, with some already approved for other medical uses. We consider irreversible those inhibitors that presented covalent binding to a residue in the active site of the protease. Further research is required to confirm their efficacy in clinical settings for viral infections like SARS-CoV-2 and influenza.

**Table 3:** Summary of clinical trials with TMPRSS2 inhibitors.

NCT Number	Status	Study Title	Conditions	Interventions
NCT04652765	Terminated	Camostat With Bicalutamide for COVID-19	COVID-19	Drugs: Camostat Mesilate, Bicalutamide
NCT04608266	Terminated	CAMOVID: Evaluation of Efficacy and Safety of Camostat Mesylate for the Treatment of SARS-CoV-2 Infection	COVID-19	Drug: Camostat Mesylate
NCT04406389	Terminated	Anticoagulation in Critically Ill Patients With COVID-19 (The IMPACT Trial)		Drugs: Enoxaparin sodiu, Fondapariniux
NCT00589472	Completed	Androgen Deprivation Therapy and Vorinostat Followed by Radical Prostatectomy in Treating Patients With Localized Prostate Cancer	Prostate Adenocarcinoma	Drugs: Bicalutamide, Goserelin Acetate
NCT04681430	Completed	Reconvalescent Plasma/Camostat Mesylate Early in SARS-CoV-2 Q-PCR (COVID-19) Positive High-risk Individuals	COVID-19	Biological: Convalescent plasma Drug: Camostat Mesylate
NCT04729491	Completed	EAT-DUTA AndroCoV Trial	COVID-19	Dutasteride 0.5 mg, Drug: Azithromycin, Nitazoxanide
NCT01075308	Completed	SB939 in Treating Patients With Recurrent or Metastatic Prostate Cancer	Prostate Cancer	Drug: HDAC inhibitor SB939
NCT04516850	Completed	HSD3B1 Gene Polymorphisms With Outcomes in SARS-CoV-2 Infected Patients	COVID-19	Genetic: Expression of receptors and activating proteases, Genetic: Polymorphism of the HSD3B1
NCT01858441	Completed	Pharmacogenetic Study in Castration-resistant Prostate Cancer Patients Treated With Abiraterone Acetate	Pharmacogenetic Study	Drug: Abiraterone Acetate
NCT01653925	Active, not recruiting	Molecular Mechanisms of Dutasteride and Dietary Interventions to Prevent Prostate Cancer and Reduce Its Progression	Prostatic Neoplasms, Low Grade Prostate Cancer	Other: Dietary intervention first, Drug: (Dutasteride) intervention first
NCT04470544	Recruiting	Camostat Mesilate Treating Patients With Hospitalized Patients With COVID-19	Severe Acute Respiratory Syndrome	Drug: Camostat Mesilate, Other: Standard of Care
NCT04352400	Recruiting	Efficacy of Nafamostat in COVID-19 Patients (RACONA Study)	COVID-19	Drug: Nafamostat Mesilate
NCT05797597	Recruiting	Long-term Aspirin Therapy as a Predictor of Decreased Susceptibility to SARS-CoV-2 Infection in Aspirin-Exacerbated Respiratory Disease	AERD—Aspirin Exacerbated Respiratory Disease	Drug: Aspirin 300 Mg Oral Tablet
NCT04473053	Recruiting	DEFINE—Evaluating Therapies for COVID-19	COVID-19	Drugs: Nafamostat Mesilate, TD139, Other: Standard care
NCT03903835	Recruiting	ProBio: A Biomarker Driven Study in Patients With Metastatic Prostate Cancer	Metastatic Castration-resistant Prostate Cancer, Metastatic Hormone-Sensitive Prostate Cancer	Drugs: Enzalutamide Oral Capsule, Abiraterone Oral Tablet, Carboplatin
NCT04578236	Unknown status	Efficacy of Aerosol Combination Therapy of 13 Cis Retinoic Acid and Captopril for Treating COVID-19 Patients Via Indirect Inhibition of Transmembrane Protease, Serine 2 (TMPRSS2)	COVID-19	Combination Product: Aerosolized 13 cis retinoic acid plus Inhalation administration by nebulization captopril 25 mg, Drug: Standard treatment
NCT04340349	Unknown status	Low-dose Hydroxychloroquine and Bromhexine: a Novel Regimen for COVID-19 Prophylaxis in Healthcare Professionals	COVID-19	Drugs: Hydroxychloroquine Sulfate, Bromhexine 8 MG
NCT04385836	Unknown status	Trial of Alpha One Antitrypsin Inhalation in Treating Patient With Severe Acute Respiratory Syndrome Coronavirus 2 (SARS-CoV-2)	COVID-19	Drug: alpha one antitrypsin inhalation
NCT04639440	Unknown status	Impact of Adipose Tissue in COVID-19	COVID-19	Other: Adipose tissue
NCT04355026	Unknown status	Use of Bromhexine and Hydroxychloroquine for Treatment of COVID-19 Pneumonia	COVID-19	Drugs: Bromhexine Oral Tablet and/or hydroxychloroquine tablet
NCT04321096	Unknown status	The Impact of Camostat Mesilate on COVID-19 Infection	COVID-19	Drug: Camostat Mesilate,
NCT04457609	Unknown status	Administration of Allogenic UC-MSCs as Adjuvant Therapy for Critically-Ill COVID-19 Patients	COVID, Pulmonary Infection, SARS-CoV2	Drugs: Oseltamivir, Azithromycin, Biological: Umbilical Cord Mesenchymal Stem Cells
NCT04577378	Unknown status	Efficacy and Safety of Drug Combination Therapy of Isotretinoin and Some Antifungal Drugs as A Potential Aerosol Therapy for COVID-19	COVID-19	Drug: Isotretinoin (Aerosolized 13 cis retinoic acid) plus Aerosolized Itraconazole
NCT04854343	Unknown status	SLPI for Prostate Cancer	Prostate Cancer, Prostatic Neoplasm	Diagnostic Test: Secretory leukocyte protease inhibitor (SLPI) in prostate cancer, Diagnostic Test: Determination of molecular alterations, Diagnostic Test: SLPI Healthy
NCT04527133	Unknown status	An Open Non-comparative Study of the Efficacy and Safety of Aprotinin in Patients Hospitalized With COVID-19	COVID-19	Drug: Aprotinin
NCT04836806	Withdrawn	Cetirizine and Famotidine for COVID-19	COVID-19	Drug: Cetirizine and Famotidine

## Data Availability

No new data were created or analyzed in this study.
